# Establishment of a primary renal lymphoma model and its clinical relevance

**DOI:** 10.3389/fonc.2023.1089187

**Published:** 2023-08-28

**Authors:** Xiaoxi Li, Minyao Deng, Chenxiao Zhang, Lingli Luo, Hui Qian

**Affiliations:** Department of Laboratory Medicine, School of Medicine, Jiangsu University, Zhenjiang, Jiangsu, China

**Keywords:** primary renal lymphoma, extranodal lymphoma, extranodal dissemination, MA-K, MCD subtype, LymphGen, aggressive B-cell lymphoma, translation pathway

## Abstract

Extranodal dissemination is an important feature of aggressive B-cell lymphoma. Owing to the lack of available animal models, the study on extranodal dissemination of lymphoma is greatly limited. Here, we identified a novel cell line, named MA-K, which originated from the Eμ-Myc;Cdkn2a^−/−^ cell line, named MA-LN in this study. Compared to MA-LN, MA-K tended to disseminate in the kidney rather than the lymph nodes in the lymphoma transplantation model, resembling human primary renal lymphoma. The transcriptome analysis revealed that MA-K had undergone transcriptional evolution during the culture. The specialized transcriptional pattern analysis we proposed in this study identified that the FOXO1-BTG1-MYD88 pattern was formed in MA-K. Further analysis found that the translation pathway was the most enriched pathway in specially expressed genes (SEGs) in MA-K. Among the SEGs, three upregulated genes, RPLP2, RPS16, and MRPS16, and five downregulated genes, SSPN, CD52, ANKRD37, CCDC82, and VPREB3, in MA-K were identified as promising biomarkers to predict the clinical outcomes of human DLBCL. Moreover, the joint expression of the five-gene signature could effectively predict clinical outcomes of human DLBCL in three groups. These findings suggested that the MA-K cell line had strong clinical relevance with human aggressive B-cell lymphoma. Moreover, the MA-K primary renal lymphoma model, as a novel syngenetic mouse model, will be greatly useful for both basic research on lymphoma dissemination and preclinical efficacy evaluation of chemotherapy and immunotherapy.

## Introduction

B-cell lymphoma is a B-lymphoid hyperplasia disease with high heterogeneity. While most lymphomas primarily present in lymph nodes, extranodal dissemination of lymphoma is a common clinical feature observed in most subtypes of Non-Hodgkin’s B-cell lymphoma (B-NHL), including diffused large B-cell lymphoma (DLBCL, NOS) (1, 2), Burkitt’s lymphoma (BL) (3, 4), and high-grade B-cell lymphoma (HGBL) (5, 6). The disseminated organs include the central nervous system (CNS) (7), skin (8), and uterus (9). Primary renal lymphoma (PRL) is a rare malignant lymphoma and most of the PRL cases are DLBCL (10). Patients with extranodal lymphoma, such as CNS lymphoma, often have poor clinical outcome. Classification of DLBCL based on transcriptional profile (11) and genetic variation (2) links the extranodal lymphoma to the activated B-cell-like (ABC) subtype and the MCD (including MYD88^L265P^ and CD79B mutations) subtype. However, because of the lack of available animal models, the genetic and non-genetic factors of extranodal lymphoma are still unclear.

The Eμ-Myc transgenic mouse is a well-established spontaneous B-cell lymphoma mouse model (12) resembling the translocation of oncogenic Myc to the enhancer of immunoglobulin heavy (IgH) μ gene in human BL. Unlike human BL and DLBCL that originated from the mature B stage, the later stage of B-cell differentiation, Eμ-Myc lymphoma mainly originates from the pro-B and pre-B stage. Hence, Eμ-Myc transgenic mouse is not an ideal model to resemble the aggressive phenotype of human B-cell lymphoma, such as extranodal dissemination. In the combined Eμ-Myc transgenic mouse and genetically engineered modified mouse (GEMM) models, the knockout of tumor suppressor genes (TSGs), such as p53 and Arf (13, 14), could significantly accelerate lymphomagenesis and shorten survival time. Transcriptome analysis on a large cohort of Eμ-Myc transgenic mice (15) revealed that the onset of Eμ-Myc lymphoma dramatically varied and BL-like and DLBCL-like transcriptional characteristics were identified in early-onset and late-onset lymphoma, respectively. In addition, genomic analysis (16) identified that disruptive mutations in Bcor contributed to spontaneously lymphomagenesis of Eμ-Myc transgenic mouse. Given that lymph node is still the major disseminated site in most Eμ-Myc-based mouse models, understanding the mechanism of extranodal dissemination is still difficult and challenging.

Because of ease of establishing a syngeneic lymphoma transplantation model, a type of the GEM-derived allograft (GDA) model (17), the Eμ-Myc;Cdkn2a^−/−^ cell line, which usually gives rise to lymphoma in lymph nodes, is widely used for *in vivo* efficacy evaluation (18, 19). In this study, we reported a Eμ-Myc;Cdkn2a^−/−^ derived cell line that could give rise to extranodal lymphoma, specifically to kidney, in a GDA model. To distinguish it from the parental Eμ-Myc;Cdkn2a^−/−^ cell line, we named the kidney-disseminated cell line as the MA-K cell line, in which the M referred to Myc and the A referred to Arf. Because of the strong clinical relevance of extranodal dissemination and aggressive B-cell lymphoma, we further analyzed the transcriptome profile of MA-K and explored prognostic biomarkers of human DLBCL inspired by the transcriptome of MA-K. Translation pathway was the most enriched pathway in SEGs in MA-K. Eight SEGs in MA-K, RPLP2, RPS16, MRPS16, SSPN, CD52, VPREB3, CCDC82, and ANKRD37, were identified as promising prognostic biomarkers of human DLBCL. Together, we report that a novel MA-K cell line with renal tropism in the GDA model has strong clinical relevance with aggressive DLBCL. The MA-K GDA model will be applied to explore the genetic and non-genetic mechanism of PRL, as well as to evaluate the preclinical efficacy of chemotherapy and immunotherapy.

## Materials and methods

### Cell lines

The *Eμ-Myc;Cdkn2a* ^−/−^ cell line, also named MA-LN in this study, was a kind gift from Prof. Michael Hemann at MIT in 2011 and preserved in the laboratory of Prof. Hai Jiang at CEMCS, CAS. The MA-K cell line was established from the *Eμ-Myc;Cdkn2a* ^−/−^ cell line in our laboratory. The *Eμ-Myc;Cdkn2a* ^−/−^ cell line and the MA-K cell line were cultured in 45% DMEM, 45% IMDM, and 10% fetal bovine serum (Biosera, FB-1058), supplemented with 100 U/ml penicillin and streptomycin, and 25 μM β-mercaptoethanol.

### Lymphoma transplantation model

All mice were housed in a specific pathogen-free environment at the Laboratory Animal Research Center in Jiangsu University and treated in strict accordance with protocols, which were approved by the Animal Care and Use Committee of Laboratory Animal Research Center, Jiangsu University.

Six-week C57BL/6JGpt female mice were purchased from the GemPharmatech (Nanjing, CN). A total of 10^6^ MA-LN cells or MA-K cells in 200 μl of DPBS were injected into C57BL/6 female recipient mice *via the* tail vein. Recipient mice transplanted with the MA-LN cell line usually grew a palpable mass at axillary lymph nodes at the 4th week post-transplantation. Instead, typical symptoms, including hunch back, dull hair, movement retardation, and abdominal bulge, were usually observed in MA-K recipient mice at the 4th week post-transplantation. Recipient mice transplanted with MA-LN or MA-K were monitored until any one of the above-mentioned symptoms arose and were sacrificed for evaluating lymphoma dissemination.

### H&E staining and imaging

For histological H&E staining, lymphomas were fixed in 10% formalin overnight and subsequently transferred into 70% ethanol, embedded in paraffin according to standard protocols. Sections (8 μm) were stained with H&E and images from the whole slide were acquired by Pannoramic MIDI (3DHISTECH) and analyzed by CaseViewer software (3DHISTECH).

### RNA extraction and RNA sequencing

Two replicates of MA-LN and MA-K, collected at different times, were applied to RNA sequencing. Total RNA was extracted using the Trizol reagent kit (15596018, Invitrogen) according to the manufacturer’s protocol. RNA library construction and sequencing were performed by Gene Denovo Biotechnology Co. (Guangzhou, China). The enriched mRNA by Oligo(dT) beads was fragmented into short fragments using fragmentation buffer and reversely transcribed into cDNA by using NEBNext Ultra RNA Library Prep Kit for Illumina (#7530, New England Biolabs). The purified double-stranded cDNA fragments were end repaired, A base added, and ligated to Illumina sequencing adapters. The ligation reaction was purified with the AMPure XP Beads (1.0×). Ligated fragments were subjected to size selection by agarose gel electrophoresis and polymerase chain reaction (PCR) amplified. The resulting cDNA library was sequenced using Illumina Novaseq6000.

### Specially expressed genes analysis

For each transcription region, an FPKM (fragment per kilobase of transcript per million mapped reads) value was calculated to quantify its expression abundance and variations, using RSEM software.

To filter SEGs with biological significance, we divided the gene expression level into three levels as FPKM ≥ 10, FPKM ≥ 1, and FPKM < 1. Inactive genes or basal expressed genes were defined as FPKM < 1. Active genes were defined as FPKM ≥ 1. SEGs were filtered as follows. Inactive genes (FPKM < 1) in both groups were directly excluded for SEGs analysis. For active genes (FPKM ≥ 1) in both groups, genes with Log_2_(FC) ≤ −2 and Log_2_(FC) ≥ 2 were filtered as SEGs. In the case of inactive genes (FPKM < 1) in one of the groups, active genes (FPKM ≥ 10) in another group were directly listed into SEGs. All SEGs were listed in **Data Sheet 1**.

Pathway enrichment analysis and protein–protein interaction (PPI) enrichment of SEGs were performed with Metascape (https://metascape.org). PPI enrichment analysis had been carried out with the following databases: STRING and BioGrid. Only physical interactions in STRING (physical score > 0.132) and BioGrid were used.

### Survival analysis

Survival analysis was performed with the online tool SurvExpress (20). A human DLBCL dataset [Lenz Staudt Lymphoma GSE10846 (21), *n* = 420] was chosen for survival analysis. The prognostic index (PI) was calculated by the expression value and the Cox model to generate the risk groups. The optimization algorithm was applied in risk grouping. The SurvExpress program was performed according to the tutorial.

### Statistical analysis

Depending on the type of experiment, log-rank test or *f*-test was used as indicated in figure legends. *p*-values <0.05 were considered significant (* < 0.05, ** < 0.01, *** < 0.001).

## Results

### Establishment of primary renal lymphoma model

**Figure 1:**
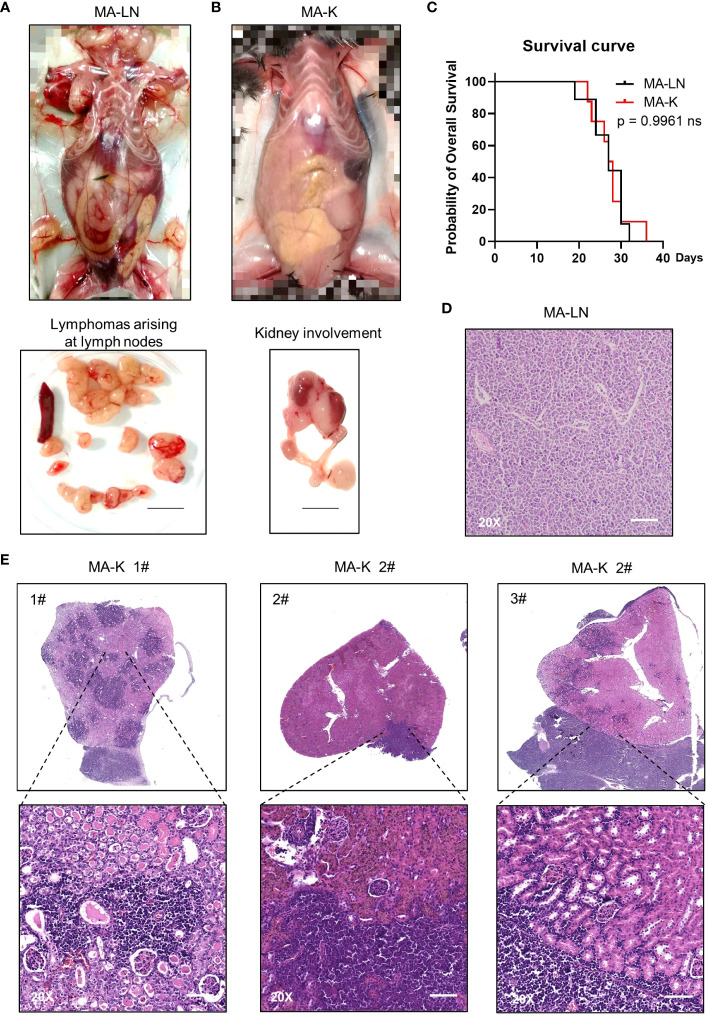
MA-K tends to disseminate in the kidney of recipient mice in the lymphoma transplantation model. **(A)** A representative picture showing the disseminated sites of MA-LN lymphoma in recipient mouse. The lymphomas were dissociated from mandibular lymph nodes, axillary lymph nodes, and inguinal lymph nodes of the recipient mice. Spleen was not enlarged. Bar, 1 cm. **(B)** A representative picture showing the unaffected lymph nodes and affected kidney and ureter in MA-K recipient mice. Bar, 1 cm. **(C)** Kaplan–Meier plots of MA-LN and MA-K recipient mice. MA-LN, *n* = 9; MA-K, *n* = 8. The equality of survival curves was tested using a log-rank test. **(D)** A representative H&E staining section of MA-LN lymphoma. Bar in 20× image, 200 μm. **(E)**. Representative H&E staining sections of primary renal lymphoma arising in MA-K recipient mice. Bar in 20× image, 200 μm.

The Eμ-Myc;Cdkn2a^−/−^ cell line, also called MA-LN in this study, is a cell line widely used to establish the lymphoma transplantation model, a type of GDA model. M refers to Myc gene and A refers to Arf gene. MA-LN lymphoma typically presented in lymph nodes (LNs) in recipient mice (**Figure 1A**), and the progression of lymphoma could be well monitored by touching the palpable mass arising in the axillary lymph nodes.

The MA-LN cell line was first introduced to establish the MA-LN GDA model in 2011. In 2021, we began to notice some obvious symptoms that we had never seen before, including hunch back, dull hair, movement retardation, and abdominal bulge, instead of palpable mass at LNs. Anatomical results showed that lymphoma was mainly disseminated at the kidney and LNs and spleens were no longer involved (**Figure 1B**), which was highly similar to human PRL. Despite the differences of disseminated sites, there was no difference in survival time of MA-LN and MA-K recipient mice (**Figure 1C**).

Considering that we had changed the source of recipient mice, we suspected that the source difference of recipient mice probably contributed to kidney dissemination of lymphoma. Hence, we successively replaced recipient mice from three different sources. The results showed that lymphoma was still disseminated at the kidney.

A study (22) had proven that cancer cell lines could evolve in culture, forming genetic and transcriptional heterogeneity and different drug responses. Therefore, we proposed that the Eμ-Myc;Cdkn2a^−/−^ cell line had evolved into a novel and stable cell line, renamed as MA-K, indicating the tendency of MA-K to kidney dissemination in the GDA model.

The histological analysis of MA-LN lymphoma showed that the lymphoma mass was mainly composed of lymphoma cells (**Figure 1D**). For MA-K lymphoma, we analyze several affected kidneys and typical H&E staining sections were presented (**Figure 1E**; **Supplementary Image 1**). According to HE staining images, we found that MA-K lymphoma can infiltrate kidneys from both renal glomerulus (#1) and renal capsule (#2 and #3). Because of the lack of *in vivo* tracing analysis of pathological progression, we cannot describe the detailed process of kidney dissemination. We speculated that MA-K lymphoma first infiltrated from glomerulus, and then the oversized lymphoma on the outer surface of the kidney could invade the kidney from the renal capsule.

### MA-K had specialized transcriptional patterns and abnormal expression of LymphGen

To identify the molecular characteristics of MA-K cells, we performed RNA-Seq analysis and 12,809 genes were initially detected in MA-LN and MA-K. To obtain differentially expressed genes (DEGs) with biological significance, we removed genes with FPKM < 1 and 8,547 genes were left. Surprisingly, approximately 20% of genes (1,905 of 8,547) had differentially expressed more than twice, indicating that MA-K was completely different from MA-N at the transcriptional level. Given that cancer cell lines could transcriptionally evolve in culture (22), we attributed the huge transcriptional difference to transcriptional selection and adaptation during the culture.

**Figure 2:**
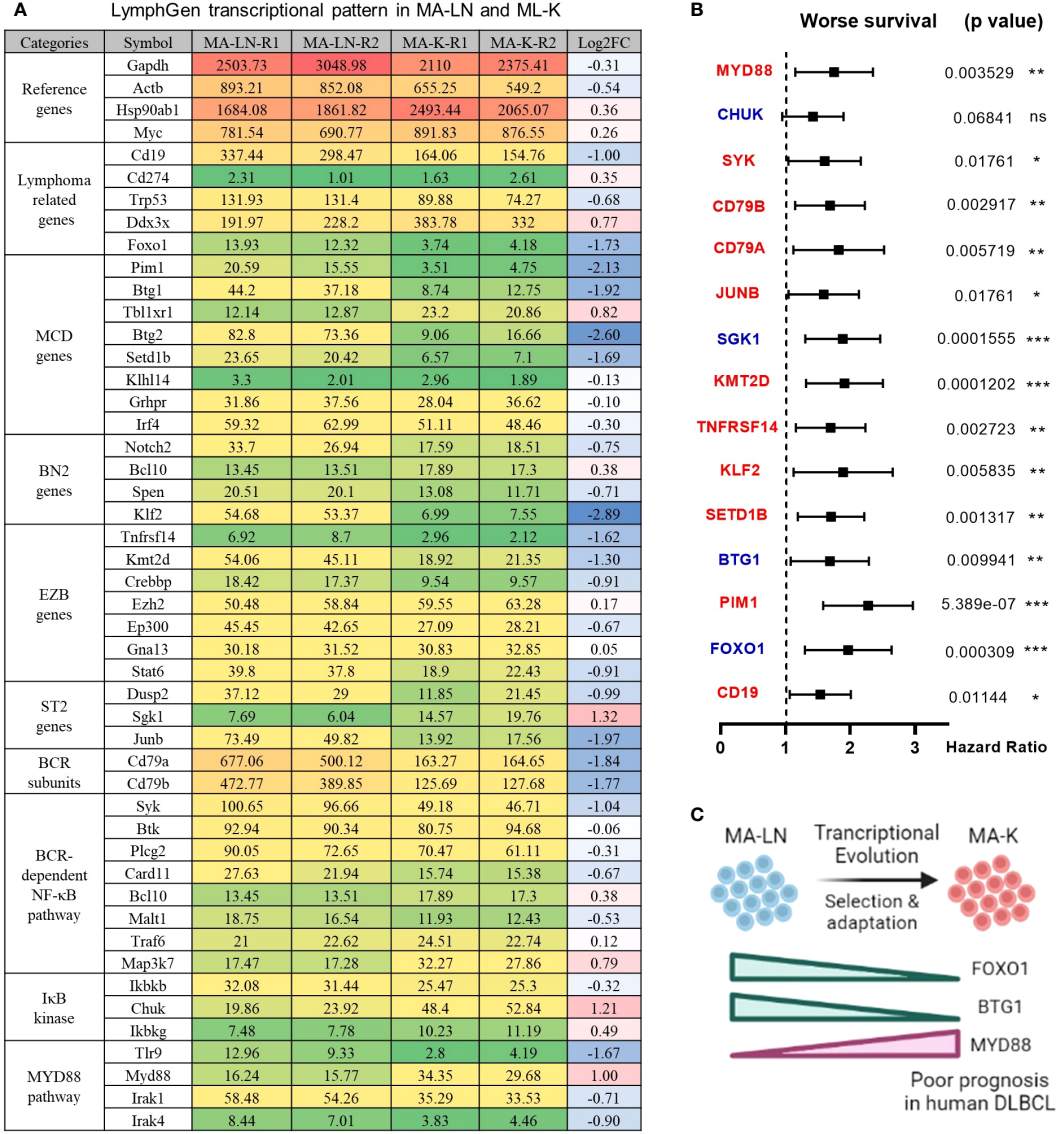
LymphGen signature in specialized transcriptional pattern of MA-K and clinical relevance. **(A)** Expression level of LymphGen genes in MA-LN and MA-K. FPKM were used to evaluate the gene expression abundance in MA-LN and MA-K. The reference gene panel including Gapdh, Actb, Hsp90ab1, and Myc was presented to the quality and comparability of FPKM data. Log_2_(FC) was calculated by the average FPKM. FC, fold change. **(B)** Forest plot of indicated genes in two risk groups. *p*-values of the log-rank test were shown. The hazard ratio (HR), confidence interval, and *p*-value in forest plot were obtained from the SurvExpress program. **(C)** Diagram for the formation of FOXO1-BTG1-MYD88 pattern during the evolution of MA-K. Created with BioRender.com. A p-value< 0.05 was regarded as statistically significance. (* < 0.05, ** < 0.01, *** < 0.001). ns, no significance.

To identify the molecular patterns of MA-LN and MA-K, we proposed the specialized transcriptional pattern (STP) and specially expressed genes (SEGs), instead of routinely DEGs, to describe the molecular pattern of the individual sample. Four reference genes, Gapdh, Actb, Hsp90ab1, and Myc, were used as the reference gene panel to determine the quality and comparability of FPKM data. A total of 44 LymphGen genes were selected to perform STP analysis. Owing to basic expression level (FPKM < 1), Bcl2, Bcl6, Bcl10, and other LymphGen genes were not included in the panel of 44 LymphGen genes. Compared to MA-LN, 14 downregulated SEGs and 3 upregulated SEGs [log_2_(FC) ≤ 1 and log_2_(FC) ≥ 1] in MA-K were identified in 44 LymphGen genes (**Figure 2A**). The observation indicated that gene inactivation by transcriptional inhibition was happening during the evolution of MA-K, which is consistent with high-frequency inactivation mutations in human B-NHL.

To test whether the gene expression level of SEGs in MA-K could predict clinical outcome, we performed survival analysis using a human DLBCL dataset (Lenz Staudt Lymphoma GSE10846, *n* = 420). We assumed that the STP of MA-K was associated with poor prognosis. The SurvExpress program was used to validate if the gene expression status could predict prognosis, in which the expression data of a single gene or multiple genes was calculated to the risk score, also called the prognostic index (PI) (20). The human DLBLC dataset (Lenz Staudt Lymphoma GSE10846, *n* = 420) was chosen, which contained detailed clinical information and had been adopted by many studies.

The results showed that most genes were associated with clinical relevance, while most of the gene expression level in risk groups was contrary to expectations (**Figure 2B**). Only FOXO1, BTG1, and MYD88 were in line with expectations (**Figure 2C**), indicating that the abnormal transcriptional pattern for specialized lymphoma was very complicated due to the heterogeneity of lymphoma. Although the significance of transcriptional evolution of MA-K was not fully understood, most LymphGen genes were indeed significantly altered at the transcriptional level.

### Translation pathway is altered in MA-K with clinical relevance

**Figure 3:**
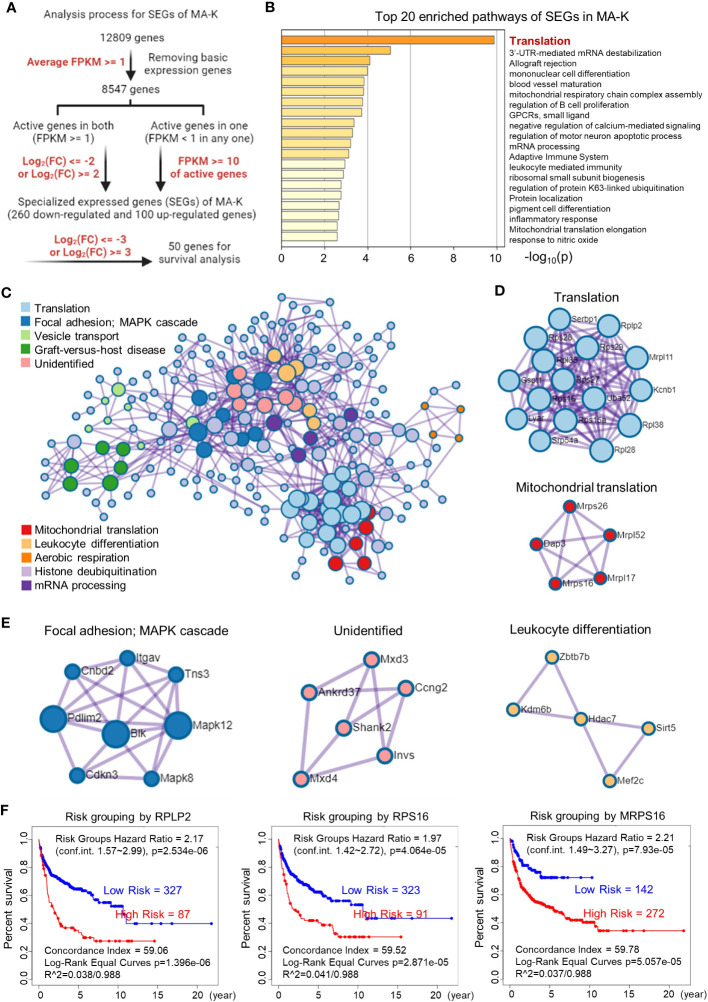
Translation-related proteins, altered in MA-K, are associated with prognosis of human DLBCL. **(A)** The analysis process of SEGs in MA-K. Filter parameters were highlighted in red. Inactive genes or basic expressed genes were defined as FPKM < 1 and excluded in SEGs analysis. Active genes were defined as FPKM ≥ 1. Created with BioRender.com. **(B)** The top 20 enriched pathways of SEGs in MA-K. In total, 360 SEGs were selected as described in Materials and methods and applied to pathway enrichment analysis. *p*-values are calculated based on the cumulative hypergeometric distribution. **(C)** The protein–protein interaction (PPI) networks of SEGs. **(D)** Genes in the PPI network including translation and mitochondrial translation. **(E)** Genes in the PPI network including Focal adhesion-MAPK, Unidentified, and Leukocyte differentiation. **(F)**. Kaplan–Meier plots of RPLP2, RPS16, and MRPS16 in human DLBCL. Red, high-risk group. Blue, low-risk group. Risk groups were generated based on the prognostic index (PI) for each gene and the optimization algorithm was applied in risk grouping. The number of each risk group was indicated in the plots. The equality of survival curves was tested using a log-rank test. A human DLBCL dataset (Lenz Staudt Lymphoma GSE10846, *n* = 420) was chosen for survival analysis.

To filter SEGs with biological significance, we analyzed the expression data as follows (**Figure 3A**). Inactive genes or basic expressed genes (defined as FPKM < 1) in both MA-LN and MA-K had been excluded in the 8,547 genes. For active genes (FPKM ≥ 1) in both, genes with log_2_(FC) ≤ 2 and log_2_(FC) ≥ 2 were filtered as SEGs. In the case of inactive genes in one, active genes (FPKM ≥ 10) in another were filtered as SEGs. All SEGs are listed in **Data Sheet 1**. A total of 360 SEGs were identified, specifically 100 upregulated SEGs and 260 downregulated SEGs in MA-K.

The pathway enrichment analysis revealed that translation pathway was the most affected pathway in SEGs (**Figure 3B**). In addition, the PPI network analysis discovered two core PPI networks in SEGs (**Figure 3C**). A core PPI network involved proteins in the translation machine, including the mitochondrial translation machine (**Figure 3D**). Another core PPI network involved proteins in Focal adhesion-MAPK, Unidentified, and Leukocyte Differentiation (**Figure 3E**). Together, the results suggested that the alteration of translation pathway and others presented the molecular features of MA-K cells.

Considering that many ribosomal proteins were abnormally regulated in tumors, we further analyzed the clinical relevance of the expression level of ribosomal proteins in human DLBCL. Survival analysis revealed that the high expression level of RPLP2, RPS16, and MRPS16, upregulated in MA-K, was significantly correlated with the poor prognosis of human DLBCL (**Figure 3F**).

Together, the transcriptional profiles discovered that translation pathway and others were largely altered in MA-K.

### Identification of the five-gene signature to predict prognosis of human DLBCL

**Figure 4:**
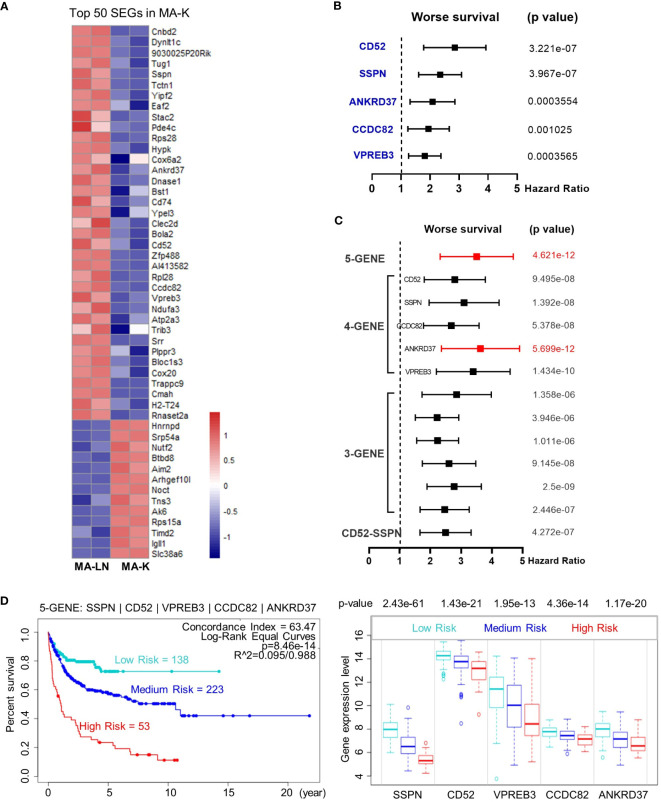
The five-gene signature, altered in MA-K, predicts the prognosis of human DLBCL. **(A)** Heatmap of SEGs in MA-K. Fifty SEGs in MA-K [log_2_(FC) ≤ 3 and log_2_(FC) ≥ 3] were analyzed. FPKM of selected SEGs were used to generate the heatmap in R studio. **(B)** Forest plot of SSPN, CD52, VPREB3, CCDC82, and ANKRD37 in two risk groups. *p*-values of the log-rank test were shown. The hazard ratio (HR), confidence interval, and *p*-value in forest plot were obtained from the SurvExpress program. **(C)** Forest plot of different combinations of SSPN, CD52, VPREB3, CCDC82, and ANKRD37 in two risk groups. *p*-values of the log-rank test were shown. The hazard ratio (HR), confidence interval, and *p*-value in forest plot were obtained from the SurvExpress program. **(D)** Kaplan–Meier plots of the five-gene signature and box plot of gene expression by three risk groups. Red, high-risk group. Cyan, medium-risk group. Blue, low-risk group. Risk groups were generated based on the prognostic index (PI) for each gene set and the optimization algorithm was applied in risk grouping. The number of each risk group was indicated in the plots. The *p*-value of gene expression by three risk groups in box plot was obtained from an *f*-test. A human DLBCL dataset (Lenz Staudt Lymphoma GSE10846, *n* = 420) was chosen for survival analysis.

Next, we investigated the clinical relevance of MA-K by evaluating the ability of SEGs in MA-K to predict prognosis of human DLBCL. Owing to too many SEGs in MA-K, we only chose 50 SEGs filtered with Log_2_(FC) < −3 or Log_2_(FC) > 3 for survival analysis (**Figure 4A**). Finally, five genes, downregulated in MA-K to MA-L, SSPN, CD52, VPREB3, CCDC82, and ANKRD37, were significantly correlated with poor prognosis of human DLBCL (**Figure 4B**).

To investigate whether the five genes had a synergistic effect on the predicted clinical outcome, we compared the hazard ratio (HR) and *p*-value of three-gene and four-gene combinations in two risk groups. Notably, the five-gene signature showed improved prognostic prediction (HR = 2.39, 95% confidence interval: 3.37 to 4.76, *p* = 4.621e-12) and a four-gene signature that removed ANKRD37 was similar to the five-gene signature, even better on HR. The results indicated that SSPN, CD52, VPREB3, and CCDC82 were independent prognostic biomarkers, and the four-gene or five-gene signature could be developed as a promising prognostic biomarker panel for human DLBCL (**Figure 4C**).

To further evaluate the usefulness of the five-gene signature, we tested its performance in three risk groups. The survival curves of high-, medium-, and low-risk groups were well stratified by the five-gene signature (log-rank equal curves *p* = 8.46e-14) and the individual genes were also significantly differential expressed in each risk group (**Figure 4D**). The *p*-value of the four-gene signature was a little worse than the five-gene signature (log-rank equal curves *p* = 1.465e-13).

We also noticed that the expression level of SSPN was the most different in three risk groups (*p* = 2.43e-61). Given that SSPN is a membrane protein, it suggests that SSPN, detectable by immune-based assay, is a promising prognostic biomarker for human DLBCL. These results not only identified the five-gene signature as a potential prognostic biomarker of human DLBCL, but also highlighted the clinical relevance between the MA-K cell model and human aggressive DLBCL.

## Discussion

This study reports a novel murine cell line, named MA-K. Compared to the parental Eμ-Myc;Cdkn2a^−/−^ cell line, called MA-LN in this study, MA-K cells tend to invade the kidney in a lymphoma transplantation model, resembling human PRL. Kidney involvement is a type of extranodal lymphoma and is associated with poor prognosis in human aggressive B-cell lymphoma. Although lymphoma arising in the kidney is rare (10), the remarkable behavior of MA-K model, in which lymphoma did not start primarily in lymph nodes in recipient mice, is a notable feature for most kinds of human primary extranodal lymphoma.

Dissemination into extranodal sites and primary extranodal localization are indeed biologically and clinically distinct scenarios. If lymphoma first presents in lymph nodes and then disseminates to extranodal tissues/organs with progression, it is not primary extranodal lymphoma, but secondary extranodal lymphoma. If lymphoma first appears in extranodal tissues/organs, this situation is considered as primary extranodal lymphoma. MA-LN cells mainly home to lymph nodes and form nodal lymphoma, while MA-K cells do not home to peripheral lymphatic organs such as lymph nodes and spleens. Therefore, we define the MA-K lymphoma model as a PRL model.

In addition, we notice that there is no difference in survival time between MA-K and MA-LN recipient mice. We assume that the survival time is determined by multiple factors, including the disease progression and therapeutic response. In this study, although there was no significant difference in survival time between MA-LN and MA-K recipient mice, the criteria for their experimental end points were largely different. The end point of MA-LN is obvious and palpable mass at lymph nodes. In this situation, the MA-LN recipient mice are in good condition. If the swollen lymph nodes are removed surgically or treated with drugs, the survival time of recipient mice should be significantly prolonged. The end point of MA-K is moribund status; at this point, the kidney damage is already very severe. In this case, it will be difficult for existing treatment interventions to extend the survival time of MA-K recipient mice.

Extranodal dissemination is also one of the key indicators for international prognostic index (IPI) in human lymphoma, indicating that extranodal dissemination indeed is associated with poor outcome in DLBCL patients. Although we currently do not know which genes/molecules are key biomarkers of extranodal lymphoma, similar to EMT markers in solid tumors, based on the features of extranodal dissemination and malignant progression we observed in MA-K model, we propose that compared to MA-LN, the MA-K cell line and PRL model are more aggressive.

The MA-K cell line originates from the MA-LN cell line and is identified as its kidney dissemination in recipient mice. Although we do not know how the MA-K cell line was formed, we can confirm that MA-K is largely different from the parent MA-LN at the transcriptional level. Given that cancer cell lines could undergo the genetic and non-genetic evolution in culture (22, 23), we attribute the formation of MA-K to transcriptional selection and adaptation (TSA). We propose that both the genetic mechanism, such as abnormal B-cell differentiation, and the non-genetic mechanism, such as cell plasticity at transcriptional selection and adaptation, are involved in the evolution of MA-K. Enlarged spleen is a common feature in Eμ-Myc and its derived lymphoma mouse models, but no enlarged spleens were observed in the MA-K recipient mice. Given that spleen and lymph nodes are both peripheral lymphoid organs, we speculate that the MA-K cell line had lost key genes that guide the homing ability of lymphoma cells to the peripheral lymphoid organs, ultimately leading to extranodal presentation.

To establish the relevance of MA-K and human aggressive B-cell lymphoma, we analyzed STP in MA-K by 44 LymphGen genes. FOXO1 is frequently mutated in the EZB-MYC+ subtype (2). BTG1 is frequently mutated in the MCD subtype. Oncogenically active mutations in MYD88 are observed in many extranodal lymphoma of DLBCL (9, 24–28) and classified into the MCD subtype (2). FOXO1, BTG1, and MYD88 are in line with expectations, indicating that MA-K shares the molecular pattern of MCD and EZB-MYC+ subtypes.

To confirm the clinical relevance of MA-K, we re-examined the SEGs in MA-K from two perspectives. In terms of signal pathway enrichment, we found that translation-related ribosomal proteins were enriched in MA-K, and the high expression of these genes was also correlated with the poor prognosis of human DLBCL. Emerging evidence suggested that dysregulation of onco-ribosomes could facilitate the oncogenic translation program and increase the risk of developing malignancy, including tumor behavior, therapeutic response, and clinical outcome (29–31). RPLP2, RPS16, and MRPS16, discovered in this study, had been reported to play oncogenic roles in various tumors (32–34). Hence, the gain of onco-ribosomes in MA-K suggests that pharmaceutical inhibition of translation may be a potential therapeutic vulnerability of aggressive B-cell lymphoma, and this strategy should be evaluated in the MA-K GDA model and clinical trials. Whether abnormal activation of ribosomal proteins contributes to extranodal dissemination of lymphoma and other aggressive phenotypes should be further explored. In terms of top SEGs, we chose the top 50 SEGs for evaluation of prognostic biomarkers. The five genes, downregulated in MA-K, were correlated with poor prognosis of human DLBCL, suggesting that these genes played negative regulation in aggressive progression of human DLBCL. Owing to the limitation of gene expression datasets with clinical information, we only evaluated the clinical relevance between MA-K and human DLBCL.

Meanwhile, CD52 and SSPN, as membrane proteins, probably directly participate in the interaction between lymphoma cells and tumor microenvironment and finally determine lymphoma dissemination. In terms of molecular classification and molecular diagnosis, we propose that CD52 and SSPN are ideal prognostic biomarkers to predict the clinical outcomes. As a specific antigen in all blast cells, CD52 had been developed as a promising therapeutic target by mono-antibody (Alemtuzumab) in many clinical trials (35–38). However, CD52 was downregulated in MA-K and the low expression of CD52 was correlated with the poor prognosis of human DLBCL, suggesting that patients could not benefit from anti-CD52 immunotherapy and even worse. If CD52 is a negative regulator for aggressive B-cell lymphoma, targeting CD52 will directly accelerate malignant transformation of lymphoma, such as extranodal dissemination. Hence, the adoption of anti-CD52 immunotherapy in clinical trials needs to be carefully reassessed.

In conclusion, the MA-K GDA model is a syngeneic lymphoma transplantation model in which lymphoma arising in recipient mice usually disseminated at the kidney, highly resembling human PRL. SEGs in MA-K reveal that MA-K has strong clinical relevance with human aggressive DLBCL and onco-ribosomes, and others, such as CD52 and SSPN, are identified as promising prognostic biomarkers in human DLBCL. Further studies on the MA-K cell line will provide more meaningful insights into the genetic and non-genetic mechanism of extranodal lymphoma. Moreover, the MA-K GDA model could be developed as a novel preclinical model of aggressive B-cell lymphoma and widely used for efficacy evaluation of chemotherapy and immunotherapy.

## Data availability statement

The datasets presented in this study can be found in online repositories. The names of the repository/repositories and accession number(s) can be found in the article/**Supplementary Material**.

## Ethics statement

The animal study was reviewed and approved by the Animal Care and Use Committee of Laboratory Animal Research Center, Jiangsu University.

## Author contributions

XL conceptualized and designed the experiments, and wrote the manuscript. HQ helped with designing the experiments and reviewing the manuscript. MD and CZ performed the mouse experiments. XL analyzed the transcriptome data. LL performed the prognostic analysis. All authors discussed the results and commented on the manuscript. XL and HQ jointly supervised the study. All authors contributed to the article and approved the submitted version.
