# Supplementary figures and images for: Establishment of a primary renal lymphoma model and its clinical relevance
Source: Front Oncol. 2023 Aug 28;13:1089187. doi: 10.3389/fonc.2023.1089187 (PMC10493870; doi:10.3389/fonc.2023.1089187)

Figure 1E

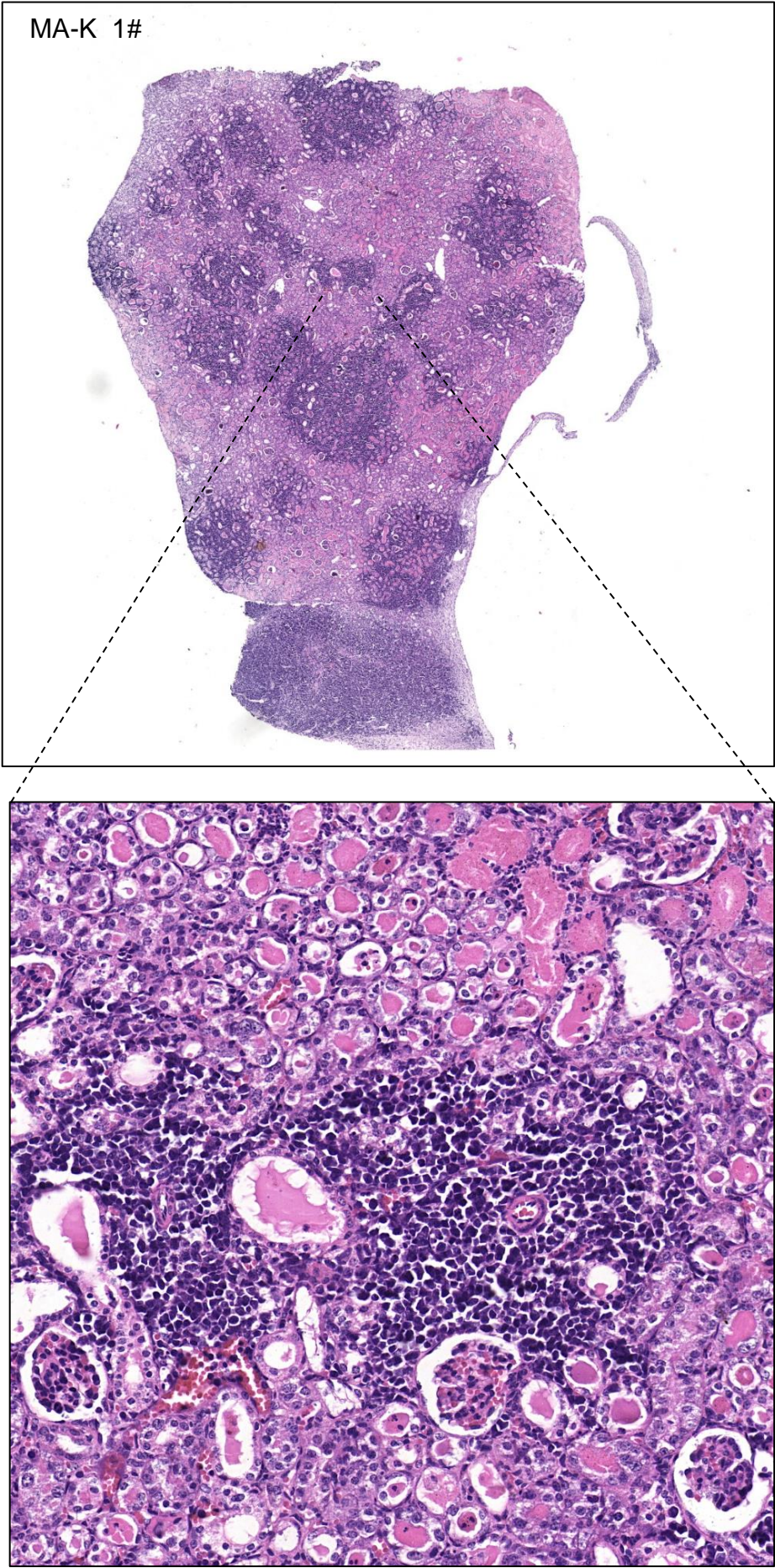

Figure 1E

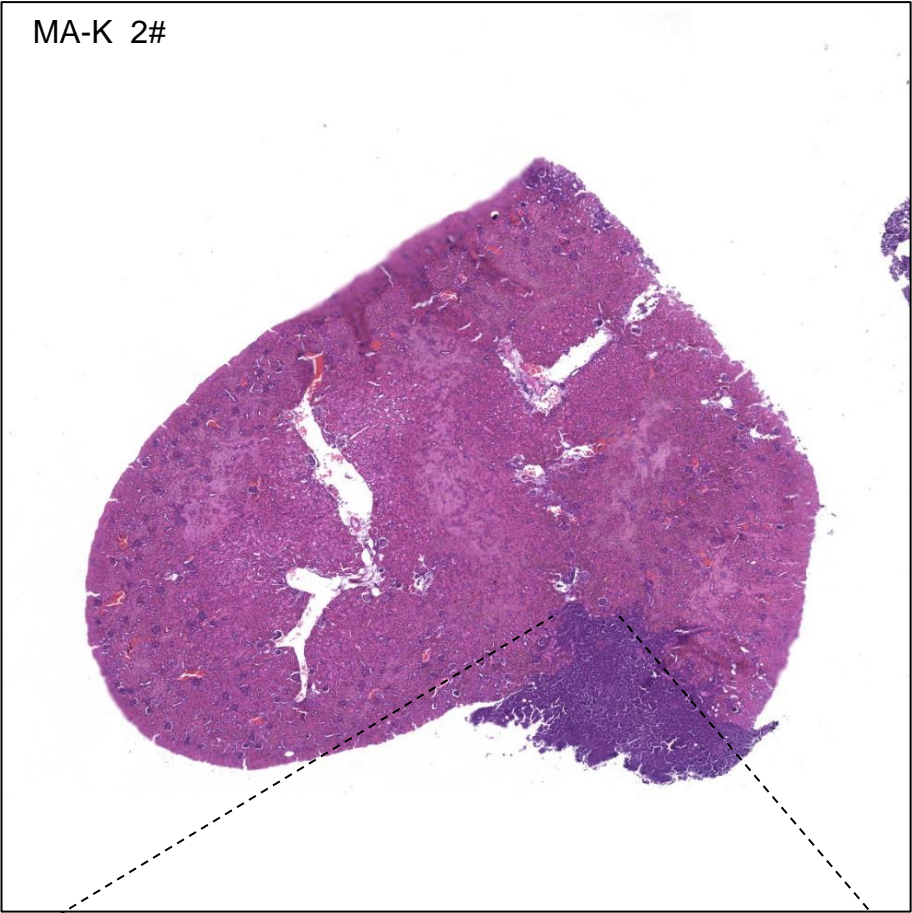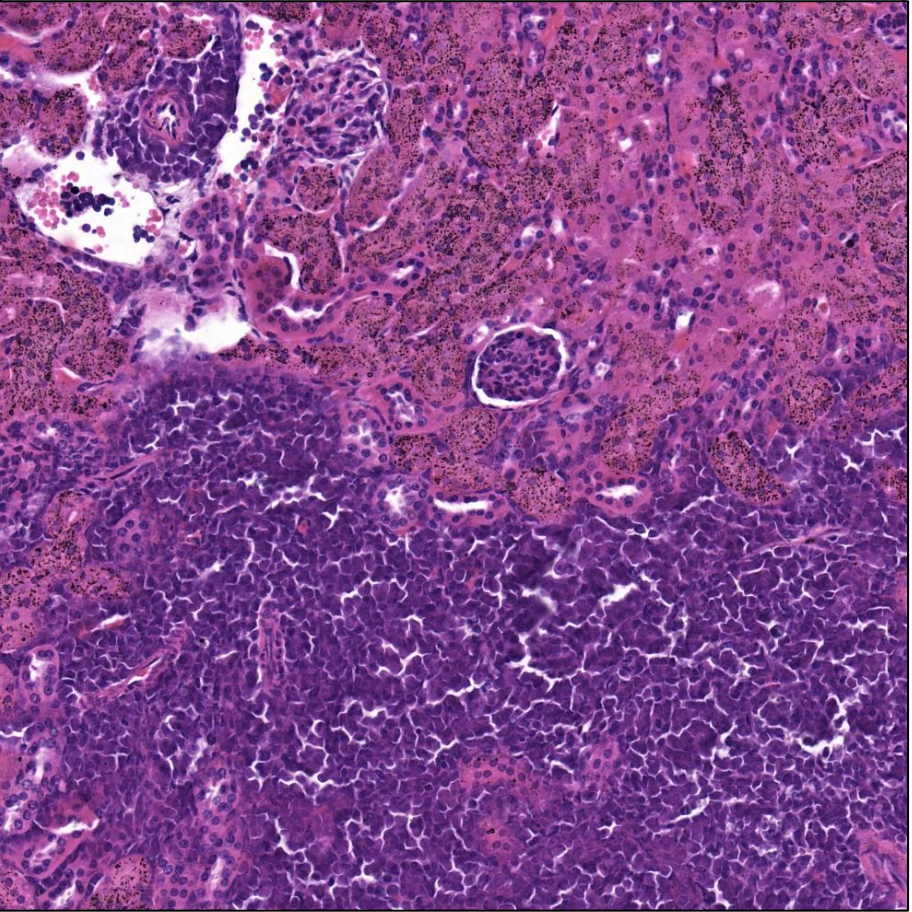

Figure 1E

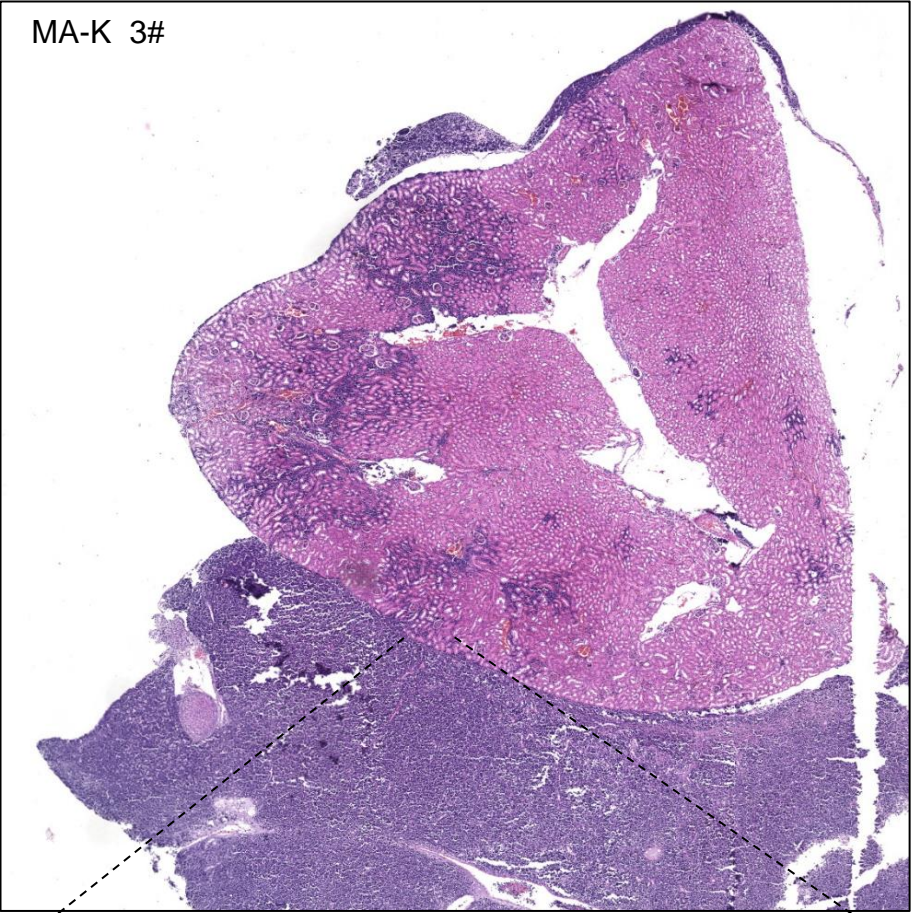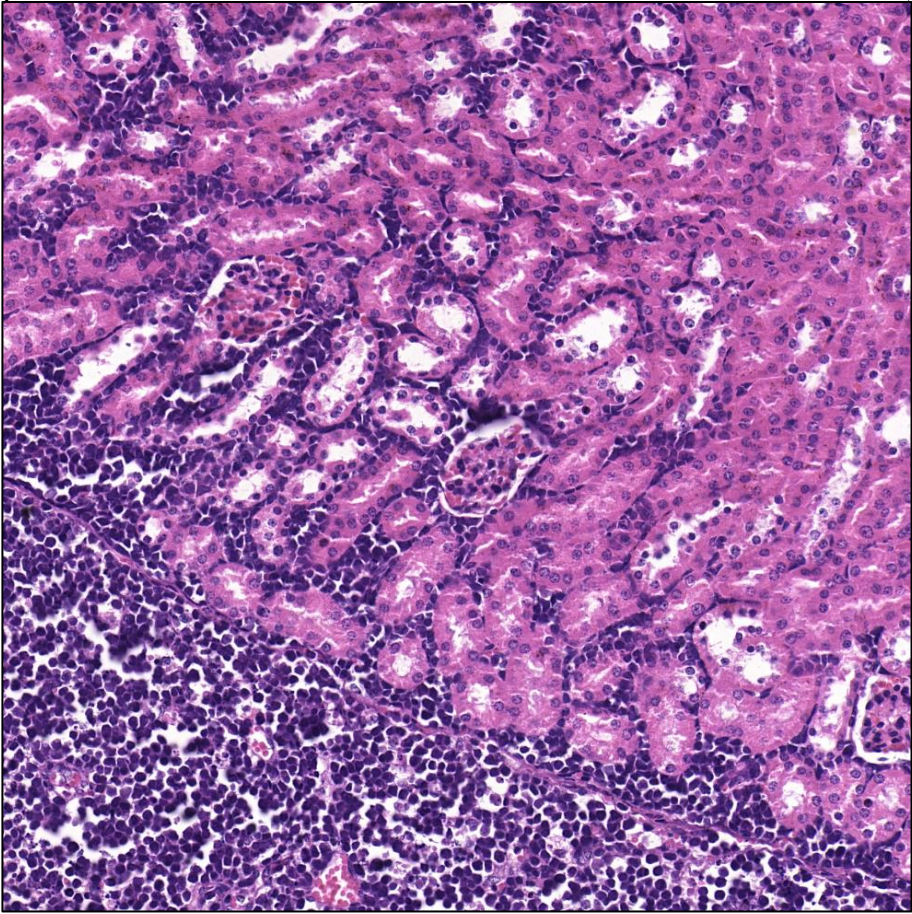

Supplement: Supplementary file 2 [file Image_1.pdf]
